# Does a root clock set the rhythm of lateral root formation?

**DOI:** 10.1093/plcell/koag253

**Published:** 2026-08-28

**Authors:** Luciano M Di Fino

**Affiliations:** Assistant Features Editor, The Plant Cell, American Society of Plant Biologists, United States; Umeå Plant Science Centre (UPSC), Department of Forest Genetics and Plant Physiology, Swedish University of Agricultural Sciences, Umeå 901 83, Sweden

One of the defining features of land plants is their ability to generate new organs after embryogenesis. Lateral roots are a prominent example of postembryonic organogenesis and play a central role in shaping root system architecture (Fig. 1a), which supports soil anchorage, water and nutrient uptake, and interactions with the surrounding environment. In *Arabidopsis*, lateral roots originate from pericycle cells located within the primary root (Fig. 1b) (De Smet et al 2007). Their formation begins with priming, a process in which semi-periodic increases in auxin signaling predispose subsets of cells to acquire competence for future lateral root formation (Moreno-Risueno et al 2010). These primed cells become lateral root founder cells and undergo coordinated divisions to generate a lateral root primordium.

**Figure 1:**
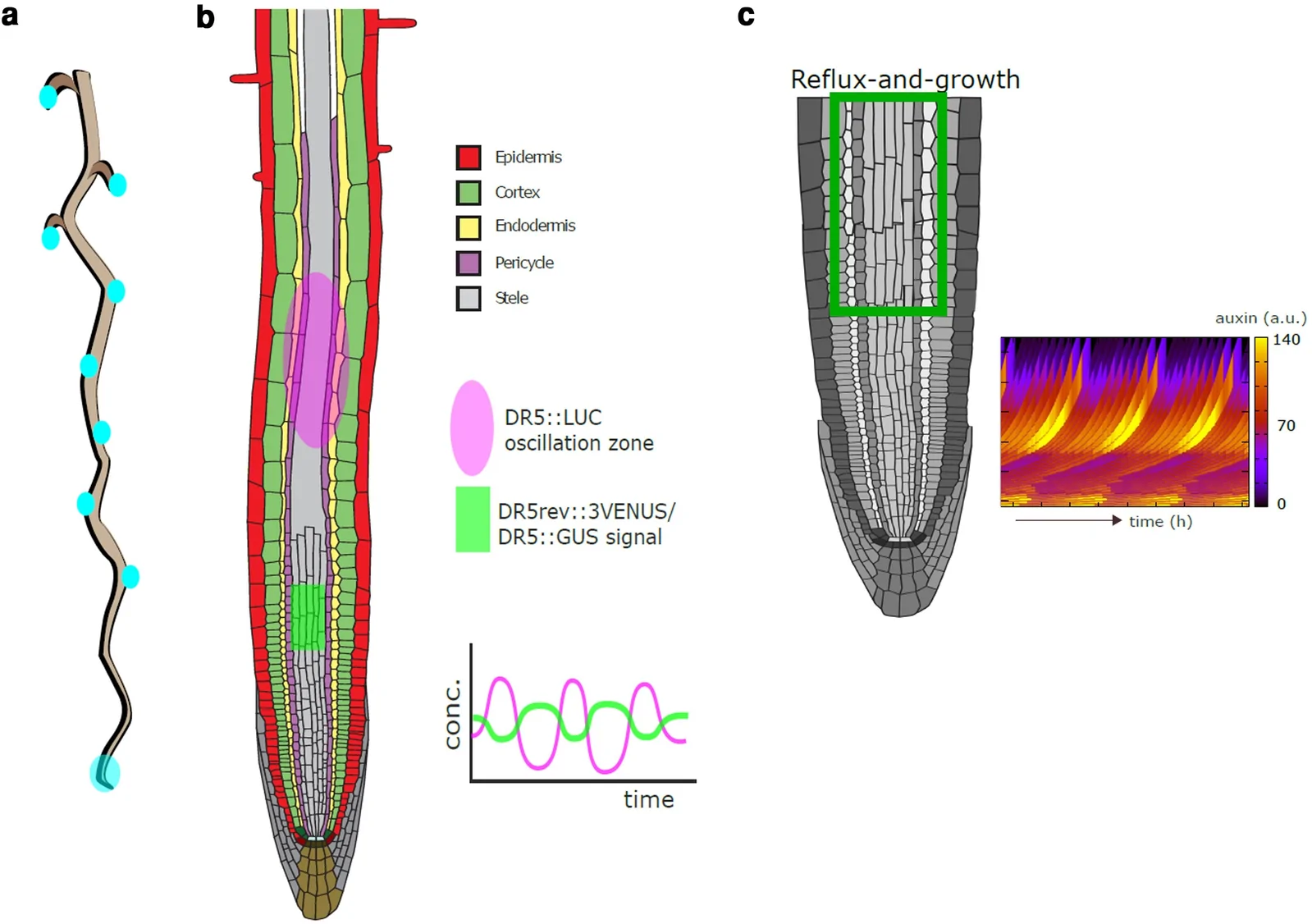
Competing models for periodic lateral root priming. a) Lateral roots form at regularly spaced positions along the primary root. Cyan dots indicate existing lateral roots and predicted sites of future lateral root formation. b) Schematic of the primary root showing the major cell types and the regions above the meristem where auxin-responsive reporters display oscillatory activity. These oscillations have been proposed to specify future sites of lateral root formation. The graph below illustrates the oscillatory behavior of these reporter signals over time. c) The reflux and growth model. Auxin reflux through the root tip creates an auxin-loading zone, while root growth and changes in cell size periodically alter auxin accumulation. The heat map below shows the resulting periodic changes in auxin levels over time within the root tissue. Adapted from Ten Tusscher (2026).

The periodic nature of auxin signaling during priming has led to the proposal of a cell-autonomous “root clock” analogous to developmental oscillators in animals (Moreno-Risueno et al 2010; Wachsman et al 2020). Biological oscillators generate repeated cycles of molecular activity and can coordinate developmental events in time and space (Novák and Tyson 2008). In roots, such a clock has been proposed to periodically modulate auxin signaling, thereby defining where future lateral roots will form. However, a key unresolved question is what generates this periodic behavior: an autonomous genetic oscillator operating within individual cells, or coordinated tissue-level processes involving auxin transport and root growth.

In this study, Ten Tusscher (2026) uses mathematical models to ask whether an internal “root clock” in individual cells can explain how lateral roots are periodically initiated. Their models suggest that auxin signaling can oscillate (Fig. 1b), but only under a limited set of conditions, and that these oscillations become progressively less robust as biological complexity is added. Oscillations can generate a regular pattern of future lateral root sites when they begin in the meristem, where dividing cells can transmit timing information to their daughter cells. However, when oscillations begin after cells enter the elongation zone, as experimental observations suggest, cells start from similar states and remain largely synchronized. Although auxin signaling continues to oscillate, the cells do not develop sufficiently different phases to generate regularly spaced auxin signaling maxima and therefore fail to specify a periodic pattern of future lateral root sites.

Periodic auxin inputs can create differences between cells but only after several cycles; in this case, the external signal rather than the internal clock sets the rhythm of pattern formation. Together, these findings argue against a cell-autonomous root clock and instead favor mechanisms that emerge from interactions across the tissue. Among these, Ten Tusscher highlights the reflux and growth model as a parsimonious alternative (Fig. 1c) in which the periodicity of lateral root priming arises from the interaction between tissue-level auxin transport and root growth, rather than from an autonomous molecular clock.

Mathematical modeling is particularly valuable in developmental biology because it allows researchers to test whether a proposed molecular mechanism can actually generate the spatial and temporal patterns observed in living tissues. Here, modeling moves the question beyond whether auxin signaling can oscillate to whether those oscillations are robust, coordinated, and positioned appropriately to explain lateral root priming. The study therefore identifies several priorities for future work: determining where oscillatory information originates, testing whether cells retain or exchange phase information across tissues, and resolving how auxin transport, cell growth, division, and root cap dynamics interact to produce periodic priming events. More broadly, this work illustrates how iterative cycles of modeling and experimentation can refine developmental hypotheses and reveal mechanisms that may remain hidden when molecular components are considered in isolation.

## Recent related articles in *The Plant Cell*

- Zhang et al (2024) investigated how salt stress reshapes lateral root development and shows that high salinity reduces auxin signaling while maintaining the activity of the key lateral root regulator LBD16 through an auxin-independent pathway.
- Dupouy et al (2026) combined live-cell imaging and mathematical modeling to investigate how root hairs switch from slow to rapid tip growth. The authors show that coordinated changes in cytoskeletons, nuclear position, and cell mechanics drive this developmental transition, and they integrate these processes into a mathematical model that reproduces key features of root hair growth.

## Data Availability

No new data was generated.
